# Assessing the Potential of a Candidate Dengue Vaccine with Mathematical Modeling

**DOI:** 10.1371/journal.pntd.0001450

**Published:** 2012-03-27

**Authors:** WHO-VMI Dengue Vaccine Modeling Group

**Affiliations:** Centers for Disease Control and Prevention, United States of America

## Background

Dengue viruses are single-stranded positive-sense RNA viruses (genus *Flavivirus*, family Flaviviridae) that are the etiological agents of dengue fever (DF). More than 2 billion people live in dengue-endemic areas [1]–[3], and dengue virus infections account for an estimated 500,000 episodes of severe disease each year [4]. A recent review suggests that these may be underestimates [5]. Despite the fact that the virus has been expanding in geographic range over the past four decades [6]–[12], there are still no licensed drugs or vaccines and no consistently effective vector interventions to combat dengue. DF is caused by four antigenically distinct viral serotypes. Each type gives rise to both life-long serotype-specific immunity and short-term cross-protective immunity against the other serotypes thought to last between 2 and 9 months [13]. The spectrum of disease ranges from asymptomatic infection to life threatening dengue hemorrhagic fever (DHF) and dengue shock syndrome (DSS). The most distinctive feature of dengue's clinical/epidemiological profile is the increased risk of severe disease following infection by a heterologous dengue serotype in an immunologically primed individual. During this secondary infection, a complex interaction is triggered between the host's immune system and the infecting virus. In this setting, elevated risk of severe dengue has been attributed to the circulation of sub-neutralizing concentrations of heterologous anti-dengue virus antibody creating an effect known as antibody-dependent enhancement (ADE) of infection and greater viral burden in vivo [2], [14]–[16]. In turn, this leads to a host immune response that is suggested to precipitate increased capillary permeability, cardiovascular shock, and hemorrhage characteristic of clinically severe dengue. Viral and other host factors may also contribute to pathogenicity. To accurately assess the effects of dengue vaccine candidates on individuals and populations, these pathophysiological mechanisms of severe dengue must be understood.

The most advanced dengue vaccine candidate—a live-attenuated, tetravalent, chimeric yellow fever dengue vaccine—commenced Phase II and Phase IIB clinical trials in 2009, and Phase III trials in December of 2010 [17]–[21]. Preliminary results have demonstrated significant immunogenicity in all age groups after three vaccine doses over a 12-month period. Immunogenicity increased steadily with each dose and was higher in individuals with previous flavivirus immunity [21]. A tetravalent dengue vaccine (TDV) candidate is currently the preferred formulation of a dengue vaccine, as it should prevent infection by all serotypes, thereby eliminating the potential risk of severe infections associated with pre-existing immunity [22].

In line with the theory behind ADE, subneutralizing antibody concentrations—theoretically occurring when immunity is waning or between vaccine doses—represent a potential risk of severe dengue to patients infected with wild-type virus during this critical period. This individual-level risk can be evaluated with sufficient follow-up, but population-level effects cannot be analyzed in the context of a vaccine trial. Population-level immunity may change the proportion of infections that occur in individuals with partial immunity, and these infections may be associated with higher viraemia and thus possibly higher transmission, generating a potential indirect detrimental effect of vaccination [23]. Although there is no evidence that vaccine-derived immunity could lead to increased severity or transmissibility upon infection, given the immunopathogenesis of dengue, this possibility should be planned for.

Population-level effects, whether related to ADE or not, can be analyzed with mathematical models. Since it is not feasible to enroll and randomize populations to dengue vaccine or placebo, mathematical models may provide the only environment where multiple types of population-wide dengue strategies can be evaluated. Models allow for assessment of multiple intervention and evaluation strategies. They can be used to understand the specific population-level mechanisms by which vaccines reduce incidence and can aid in the design of evaluation studies. The World Health Organization (WHO) has recommended that mathematical models be used to assess and inform various methods of new vaccine introductions [24], [25].

To date, most models of dengue transmission have been limited in scope and focused on specific questions in transmission dynamics. However, many aspects of the dynamics of dengue transmission are still not fully understood. In order for models to be accurate, realistic, and useful, there is an urgent need for improved understanding of dengue virology and immunology, as well as the entomological, social, and environmental factors that modulate dengue transmission. As these facets of dengue biology are further investigated, we will gain confidence that future mathematical models may come close to an accurate representation of true dengue epidemics.

## Mathematical Modeling

**Figure 1:**
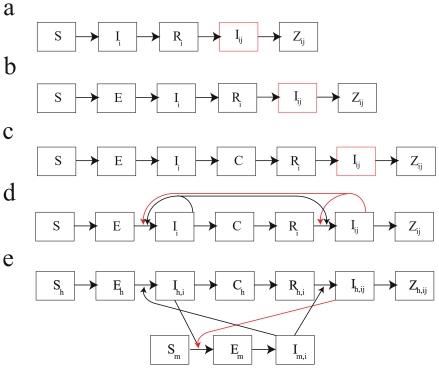
Example structures of dengue models. The disease state space of five alternative dengue model structures incorporating immune enhancement and short-term cross protection are shown. The disease states are: *S* susceptible, *E* exposed but not yet infectious, *I*_i_ infectious with serotype *i*, *I*_ij_ infectious with serotype *j* having had serotype *i*, *R*_i_ recovered from and immune to serotype *i*, *Z*_ij_ recovered from and immune to serotypes *i* and *j* and hence immune to all serotypes, *C* temporarily cross-protected from all serotypes due to recent exposure. Model (*a*): individuals immune to one serotype are more likely to experience a severe infection (denoted by red box). Model (*b*): similar to model *a* with the addition of a pre-infectious exposed class *E*. Model (*c*): includes a short-term cross-protection class *C* in which recently recovered individuals are protected from infection for a certain amount of time. Model (*d*): model with short-term cross-protection and increased infectiousness of class *I*_ij_ indicated by red arrows showing an increase in the rates of acquisition of primary and secondary infection due to this effect. Model (*e*): increased transmissibility among secondary infections *I*_ij_ to a mosquito species. Note that in this formulation, mosquitoes that have obtained infection from a secondary human infection are not more likely to transmit to humans. Subscripts *h* and *m* denote human and mosquito, respectively.

A mathematical model is a set of equations or rules describing how a certain process unfolds in time. Manipulating these rules allows one to experiment with components of the model to explore their effects on the modeled process as a whole, and it allows one to compare predicted model outcomes with observed data. Mathematical models of disease transmission have three main purposes: understanding the fundamental driving forces of disease ecology and epidemiology, measuring epidemiological parameters that cannot be directly measured with field or laboratory data, and making predictions of future disease incidence under specified conditions. Recent applied dengue modeling examples include models to explore and validate the effects of weather on the mosquito life cycle [26], to estimate serotype-specific forces of infection [27], to determine the degree to which ADE enhances viral fitness [28], to test if ADE alone is sufficient to generate the oscillating serotype patterns seen in dengue [29], [30], to determine the impact that long-term trends in dengue transmission rates may have on DHF incidence [31], to determine if long-term demographic trends are responsible for a shift in the age structure of dengue cases [32], and to investigate whether tertiary or quaternary dengue infections are compatible with the known epidemiology of dengue [33]. Although none of these models included vaccination, they provide the necessary modeling platform in which the impacts of alternative dengue control strategies, and vaccination in particular, can be evaluated. Some common dengue model structures are shown in Figure 1.

Dengue modeling has been useful in helping us understand the virus' dynamics and in generating some new hypotheses about why the dynamics exhibit certain irregularities, both short-term and long-term. Nevertheless, when compared to diseases such as influenza or malaria, the dengue modeling literature is sparse and focused on a small number of topics, often serotype oscillations or antibody-dependent enhancement. Given the importance of mosquito populations to dengue transmission, we have a relatively poor understanding of their population dynamics. In addition, dengue models are rarely analyzed with a public health goal in mind, and very little modeling has been done to evaluate dengue interventions.

In developing an appropriate mathematical model (or set of models) for dengue vaccination, the main challenge lies in resolving the complexity of interactions among host immune status, demography, vector populations, and environmental factors. A current focus of much modeling work is the strong interaction between dengue immunology and epidemiology. Through conferral of immunity, dengue epidemics generate population-wide immune profiles that subsequently determine the severity, speed, and magnitude of dengue's second pass through that same population. Typically, as a dengue epidemic progresses, surveillance focuses on case numbers and severity without recording changes in immune status; this deprives us of essential data necessary for understanding the immuno-epidemiology of dengue. One of the greatest challenges for epidemiologists and mathematical modelers alike may be determining study designs that can collect data on host immune status as efficiently and completely as possible; such data sets may allow us to describe the dynamics of population-wide immunity and its effects on future disease incidence.

Because we do not yet have a well-tested general model of dengue immuno-epidemiology, we cannot predict accurately how a TDV would alter future dengue dynamics. Mathematical modeling research must thus start by identifying realistic expectations for a TDV campaign, given a varied set of scenarios for vaccine introduction in a population. These analyses may need to evaluate if TDV rollout will have a greater impact on case numbers or severity, and if vaccination-induced shifts in the age burden have positive or negative impacts on overall disease severity.

The next challenge will be to create a set of public health objectives that will define the success of a dengue vaccination campaign. Reduced case numbers, fewer severe cases, and fewer deaths are all potential marks of success, but these three indicators may not correlate with one another, either in the population as a whole or across age classes. For example, dengue in the elderly can be complicated by comorbidities that increase the risk of severe outcomes [34], [35], and severity and mortality rates can vary among age classes [36]. Focused vaccination of children may not reduce mortality rates in adults unless herd immunity is achieved, but the level of coverage needed to reach the threshold of herd immunity has not yet been established. Balancing these objectives may prove difficult, as a vaccination campaign could potentially prevent many infections today while creating the conditions for more infections in the future. Dengue modeling may benefit from previous mathematical modeling analyses of population-level public health benefits in malaria, influenza, and nosocomial infections [37]–[39].

Because the interactions among key determinants of dengue transmission, such as environmental factors and vector biology, are not well understood, exploring the role of these determinants through modeling will require significant effort. There are still gaps in our understanding of short-term cross protective immunity [13], original antigenic sin [40]–[42], long-term serotype-specific immunity [13], [43], [44], ADE, the mode of action of the vaccine, the association of infecting serotype sequence on disease severity [44]–[47], variation in mosquito biting patterns [48]–[51], host variation in susceptibility and transmission [30], [52], [53], population and vector mobility [54], [55], and virus dynamics between dengue seasons [56]. All of these factors should have an important effect on the critical vaccination fraction—or, more precisely, the age-stratified critical vaccination fraction needed to interrupt dengue transmission—as well as the optimal design of a vaccination catch-up campaign after the vaccine is introduced.

## Dengue Vaccine Modeling Group

To address these uncertainties and to accelerate the development of mathematical models that can evaluate dengue vaccination strategies, WHO and the Vaccine Modeling Initiative (VMI) convened a group of dengue epidemiologists, clinicians, immunologists, public health officials, vaccine developers, entomologists, and mathematical modelers to discuss possibilities for assessing the population-wide impact of a tetravalent dengue vaccine. This was the first such meeting, which was hosted by WHO in late 2010. Its purpose was to establish (1) a forum for an inter-disciplinary working group to discuss the development of optimal dengue vaccination strategies, and (2) future meetings with more experts and stakeholders in dengue vaccination. The WHO-VMI Dengue Vaccine Modeling Group's first phase of collaboration has begun by linking modelers with epidemiologists, clinicians, immunologists, and vaccine developers for the purpose of conducting preliminary modeling analyses on the risks and benefits of dengue vaccination.

The initial questions identified by the group as critical in assessing a dengue vaccine are listed in Box 1. Future meetings will need to include more experts on virology, vector control, demographics, environmental change and urbanization, and economic and social aspects of dengue burden. A second meeting is being planned for 2012, the goals of which will be to (1) evaluate progress of current modeling and identify critical tests to validate models, (2) identify the areas of greatest uncertainty in dengue modeling, (3) identify key data sets, reviews, and/or meta-analyses that can aid the development of models, and (4) broaden the community of natural scientists, social scientists, and policy makers involved in research on dengue vaccination.

**Box 1. Urgent Questions for Dengue Vaccination Roll-Out**

1. Are there vaccine product profiles that could lead to increased transmission from secondary infections?
2. What changes in age distribution of primary and secondary infection are expected after vaccine introduction and mass immunization?
3. Given the demographics and force of infection in any particular setting, what is the optimal age of vaccination and/or the age-stratified critical vaccination fraction?
4. If vaccine efficacy depends on pre-existing immunity, what is the optimal age of vaccination and/or the age-stratified critical vaccination fraction?
5. Should a vaccination strategy change given geographical variation in transmission?
6. How should catch-up campaigns be implemented?
7. What immune escape or other viral evolutionary responses can be expected?
8. How should the immune system be represented in models?
9. How should individual risk profiles (i.e., the characteristics of an individual, including past infections and vaccination status, that affect the individual's risk for severe dengue) be defined and modeled?
10. How should population-level vaccine effects be monitored?

## Parameterizing Models and Data Sharing

The utility of models to assess vaccine candidates depends on the models' ability to represent transmission dynamics accurately. Measuring or estimating model parameters is therefore a critical step in constructing an accurate dengue model. Some parameters can be measured directly from epidemiological or laboratory data (duration of viraemia, mean age of first infection), while in other cases models may be used to statistically infer the impacts of certain features of transmission dynamics that cannot be measured directly (duration of cross-protective immunity [57], effect of disease severity on transmissibility). In both cases, it is critical that modelers work closely with dengue virologists and epidemiologists who understand the lab/epidemiological data and the parameter measurements. These data will be critical for the iterative process of model design and validation.

Many of the individual-level parameters concerning immunity and disease severity are ideally measured in prospective cohort studies with long-term follow up. Table 1 lists the known prospective cohort studies that contain valuable individual-level data on immune responses, differences between primary/secondary infections, asymptomatic infections, disease severity, and age burden. Equally valuable data can be obtained from natural epidemics in populations where dengue has been absent for a long time [44], [58]–[60]. Analyzing these data with mathematical models will be helpful for determining many of the individual-level parameters that are necessary for evaluating population-wide dengue vaccination. As different stages of clinical trials are completed in the next several years, their results will also be critical in improving the accuracy and validity of mathematical models.

**Table 1:** Prospective Dengue Cohort Studies.

Location	Years	Ages	Follow-Up	Population with Follow-Up	Notes	Reference
Bangkok, Thailand	1962–1964	All ages	6–11 months	1,887	Includes entomological indices and hospitalization data.	[63]
Koh Samui, Thailand	1966–1967	2–12 years	1 year	336		[64]
Yangon, Myanmar	1984–1988	2–6 years	1 year	3,579	Five separate cohorts started each year. Includes hospitalization data.	[47]
Bangkok, Thailand	1980–1981	4–16 years	6 months	1,757		[65]
Rayong, Thailand	1980–1981	4–14 years	1 year	1,056		[66]
Iquitos, Peru	1993–1996	7–20 years	2.5 years	129	No DHF/DSS found in secondary cases.	[67]
Bangkok+Khamphaeng Phet, Thailand	1994–1996	6 months –14 years	1 year	168	48 had follow-up past 180 days.	[68], [69]
Yogyakarta, Indonesia	1995–1996	4–9 years	1 year	1,837		[45]
Khamphaeng Phet, Thailand	1998–ongoing	7–11 years	2 years	2,119	Study performed in two periods: 1998–2002, 2004–2006	[70]–[72]
Bandung, West Java, Indonesia	2000–2002	18–66 years	2 years	2,536		[73]
West Jakarta, Indonesia	2001–2003	Children and adults	14 days; 6 months for cases	785	Cluster investigation enrolling contacts of known cases.	[74]
Managua, Nicaragua	2001–2003	4–16 years	1–2 years	999		[75]
An Giang, Vietnam	2004–2007	2–15 years	3 years	>3,000	Additional children recruited every year. 1,594 children had 3 years of follow-up.	[76]
Managua, Nicaragua	2004–ongoing	2–9 years	4 years	3,721	Includes entomological indices.	[77], [78]
Ho Chi Minh City, Vietnam	2006–2007	Newborns enrolled	1 year	1,244 infants		[79]
Ratchaburi, Thailand	2006–2010	3–15 years	4 years	∼3,000	Study ended.	Unpublished
Colombo, Sri Lanka	2008–2010	<12 years	2 years	800	Study ended.	Unpublished
Ho Chi Minh City, Vietnam	2009–ongoing	Newborns	1 year	∼3,000 infants		Unpublished

For all those involved—whether in epidemiology, in clinical or laboratory settings, or as modelers—it is critical that complete data sets and the analysis of that data be shared so that all the partners can come to a common understanding of the interpretation of the data. The recent meeting in 2010 sought to catalyze this effort by taking advantage of the participants' varied skills and experiences and by bringing together those scientists specializing in theory/modeling with those that have a detailed understanding of the data. Sharing and analyzing data from ongoing and past studies will be critical for building robust mathematical models of dengue. The first small step in this partnership will be the joint design and analysis of mathematical models rooted in the most recent epidemiological and laboratory data, with each collaboration including modelers and non-modelers.

## Future Challenges

In addition to sharing data sets and analyses and interpreting results, we must recognize that dengue vaccination planning will probably happen alongside vector control, social outreach and educational campaigns, multiple types of surveillance, expansion of local capacity to diagnose and manage dengue, and perhaps novel entomological approaches of reducing transmission by altering mosquito ecology or genetics [61], [62]. This broader picture of dengue control may not be easy to model mathematically, but some of these aspects will need to be evaluated in terms of their added population-level benefits to dengue vaccination. Currently, very little is known about the effectiveness of modeling social dynamics or modeling epidemics and response/intervention policies in the context of imperfect surveillance.

The mathematical models developed through the joint effort of the modeling community and dengue community will give us prediction and evaluation tools that can be used to determine optimal vaccination strategies for each endemic country. Recommendations will be discussed with national public health authorities and adapted to the requirements and realities of the host countries. When an implementation method is chosen for rolling out dengue vaccines, appropriate and timely surveillance activities should be planned so that the effectiveness of the vaccination strategy can be tested and adjusted in real time. The implemented strategy will almost certainly not be the one determined to be optimal by a mathematical model, but one that combines relevant aspects of feasibility, cost-effectiveness, political acceptability, and public health benefits. We must recognize that mathematical models are at best fallible as prediction tools and that the implementation process itself will reveal new trends and facts that can be used to improve future models and recommendations.
